# Time-Resolved Epithelial Responses to ETEC-K88 Reveal Tryptophan-Linked Protection in IPEC-J2 Cells and Strain-Specific Intestinal Injury in Mice

**DOI:** 10.3390/ani16172632

**Published:** 2026-08-22

**Authors:** Zhenguo Hu, Yuezhou Yao, Sitong Chen, Songlin Zhang, Yulong Yin, Feiyue Chen, Xiongzhuo Tang

**Affiliations:** 1 Laboratory of Animal Nutritional Physiology and Metabolic Process, Key Laboratory of Agro-Ecological Processes in Subtropical Region, Institute of Subtropical Agriculture, Chinese Academy of Sciences, Changsha 410125, China; huzhenguo22@mails.ucas.ac.cn (Z.H.); yinyulong@isa.ac.cn (Y.Y.); 2 College of Modern Agricultural Sciences, University of Chinese Academy of Sciences, Beijing 101408, China; 3 College of Animal Science and Technology, Hunan Agricultural University, Changsha 410128, China; 15828281382@163.com (Y.Y.); sx20250320@stu.hunau.edu.cn (S.C.); songinzh@stu.hunau.edu.cn (S.Z.); 4 Yuelushan Laboratory, Changsha 410128, China

**Keywords:** ETEC-K88, IPEC-J2, intestinal barrier, pigs

## Abstract

Enterotoxigenic *Escherichia coli* K88 (ETEC-K88) is a primary causative agent of post-weaning diarrhea in piglets, yet most in vitro infection models evaluate epithelial injury at a limited number of endpoint time points, failing to capture the temporal dynamics of host–pathogen interactions. Here, we established a time-resolved ETEC-K88 challenge model in IPEC-J2 cells from 0 h to 48 h. The data showed that ETEC-K88 adhesion significantly increased after 4 h and peaked after 24 h, with a critical response transition at 4–8 h characterized by coordinated changes in mRNA expression of tryptophan metabolism, tight junction, aquaporins, and Solute Carrier Transporters (SLC). Additionally, we also evaluated the protective effects of L-tryptophan supplementation in IPEC-J2 with ETEC-K88 infection and found that its addition significantly restored the disrupted gene expression related to tryptophan metabolism, transporter channels, aquaporins, and cell cycle. Finally, two different mouse strains, C57BL/6J and BALB/c mice, were challenged with ETEC-K88 to assess strain- and segment-specific intestinal responses. Although overt diarrhea was not clearly induced in mice, the ETEC-K88 fimbria receptor genes were induced in both mice strains. Additionally, both mice strains exhibited obvious intestinal histomorphological abnormalities and showed the strain- and segment-specific expression of intestinal stem cell marker genes (*Lgr5*, *SOX9*), goblet cell marker gene *TFF3*, and aquaporin genes. In conclusion, we have defined a temporal epithelial response framework for ETEC-K88 infection in IPEC-J2 cells and mice, providing a theoretical basis for developing nutritional strategies against ETEC-associated intestinal dysfunction in pig production.

## Simple Summary

Enterotoxigenic *Escherichia coli K88* (ETEC-K88) is a major bacterial pathogen responsible for post-weaning diarrhea in piglets, causing significant economic losses in the swine industry. Understanding how intestinal epithelial cells respond to ETEC-K88 infection over time is crucial for developing effective nutritional intervention strategies. In this study, we established a time-resolved infection model using IPEC-J2 cells (a porcine intestinal epithelial cell line) to track dynamic cellular responses from 0 to 48 h post-infection. We found that bacterial adhesion increased markedly after 4 h, accompanied by coordinated changes in genes involved in tryptophan metabolism, barrier function, immune responses, and water transport. Furthermore, we found that L-tryptophan addition effectively restored the expression of genes related to intestinal barrier function and water transport in ETEC-K88-infected IPEC-J2 cells. To complement the cell models, we challenged C57BL/6J and BALB/c mice with ETEC-K88 and observed strain- and segment-specific intestinal morphological changes. These findings establish a temporal framework for understanding ETEC-K88 pathogenesis and support tryptophan supplementation as a potential protective strategy, providing valuable guidance for future nutritional intervention in pig production.

## 1. Introduction

Enterotoxigenic *Escherichia coli* (*ETEC-K88*) is one of the major bacterial pathogens associated with post-weaning diarrhea in piglets [1]. ETEC-K88 expresses F4 fimbrial adhesins that promote attachment to intestinal epithelial cells, after which enterotoxins disturb epithelial ion and water transport and contribute to intestinal fluid loss [2]. In addition to secretory dysfunction, *ETEC-K88* infection has been associated with impaired epithelial barrier integrity, inflammatory activation and disruption of intestinal homeostasis.

Basically, enterotoxins are primarily classified into two types: heat-stable enterotoxins (ST) and heat-labile enterotoxins (LT). Both ST and LT are able to activate the cGMP/cAMP-PKA pathway, which in turn activates ion channels, leading to disruption of intracellular osmotic balance, water loss, and ultimately diarrhea [3,4,5]. Furthermore, ETEC-K88 infection triggers intestinal barrier injury, accompanied by reduced expression of zona occludens 1 (ZO-1) and mucin 2 (MUC2) [6]. Both microbial invasion and the disruption of microbial homeostasis triggered by *ETEC-K88* infection can activate the TLR4/NF-κB signaling pathway to induce the expression of pro-inflammatory cytokines [7,8]. Moreover, diarrhea-caused impairment of intestinal barrier function and dysbiosis of the gut microbiota enable host and microbial-derived metabolites to modulate numerous functions in other organs via the gut–brain axis, gut–muscle axis, and gut–lung axis [9,10,11,12,13].

Tryptophan (Trp) is an essential amino acid that is indispensable for maintaining host energy homeostasis and gut health. The metabolism of Trp in the intestinal epithelial cells consists of two major pathways termed the kynurenine and serotonin pathways, both of which are catalyzed by limiting enzymes [14]. Additionally, gut microbes are capable of degrading Trp into various indole derivatives that possess a variety of biological functions in the gut [15,16,17,18]. Recently, Trp-derived metabolites have been extensively investigated for their roles in diarrhea alleviation and acting as diagnostic biomarkers in humans [19,20], mice [21,22], and pigs [23]. Moreover, dietary supplementation of Trp enhances the production of neutral mucins in the colon to alleviate diarrhea-caused intestinal epithelial damage [24]. However, the connection between Trp metabolism and ETEC-K88 infection-caused pathological traits in porcine models still lacks investigation.

The porcine jejunal epithelial cell line (IPEC-J2) is widely used as an in vitro model for studying *ETEC-K88* infection and screening antimicrobial peptides, feed additives, antioxidants and other protective compounds. Previous studies have employed IPEC-J2 cells to assess *ETEC-K88*-induced adhesion, oxidative injury, apoptosis and proliferation [25]. However, many existing models rely on one or two terminal sampling points, which limits their ability to capture temporal epithelial responses during infection [26,27]. This limitation creates a practical gap: without establishing the temporal emergence of *ETEC-K88* adhesion, intestinal barrier disruption, metabolic disturbance and cell-turnover responses, it is difficult to select rational intervention windows to prevent *ETEC-K88* infection-induced diarrhea in pig production.

In this study, we established a time-gradient ETEC-K88 infection model in IPEC-J2 cells to characterize bacterial adhesion and the dynamic expression of genes related to Trp metabolism, intestinal epithelial barrier function, immune defense, proliferation and water/solute transport. Furthermore, we evaluated the effect of L-Trp supplementation on the alleviation of *ETEC-K88*-induced epithelial dysfunction in IPEC-J2 cells. Lastly, we also performed ETEC-K88 challenge assays in C57BL/6J and BALB/c mice to assess the strain- and segment-specific intestinal epithelial morphological traits. Our data will provide a theoretical basis for developing effective nutritional strategies to protect pigs against *ETEC-K88* infection-induced diarrhea and intestinal injuries.

## 2. Materials and Methods

### 2.1. Ethical Statement

All animal procedures were performed in compliance with the approval of the Institutional Animal Care and Use Committee of Hunan Agricultural University (20240206).

### 2.2. ETEC Strain

*ETEC-K88* and Green fluorescent protein (GFP)-labeled ETEC-K88 (K88, F4ac) were used for infection assays. ETEC K88-GFP was kindly provided by Wenwen Wang (College of Animal Science and Animal Medicine, Shandong Agricultural University, Taian, China). Porcine jejunal epithelial cells (IPEC-J2) and ETEC-K88 were kindly provided by Bie Tan (College of Animal Medicine, Hunan Agricultural University, Changsha, China).

Enterotoxigenic *Escherichia coli* K88 (*ETEC-K88*) was inoculated into Luria-Bertani (LB) broth (HB0128, hopebio, Qingdao, China) and incubated with shaking at 37 °C and 180 rpm until the OD_600_ value reached 0.4–0.6. Subsequently, the bacterial suspension was either diluted with phosphate-buffered saline (PBS) (CB012, CellorLab, Changsha, China) or concentrated by centrifugation at 800 rpm according to the required bacterial concentration for the experiment. The pellet was washed three times with PBS and then resuspended in an equal volume of Dulbecco’s Modified Eagle Medium (DMEM) basic (Gibco, Waltham, MA, USA). The final bacterial concentration in DMEM was adjusted to the working concentration for subsequent infection assays.

### 2.3. Cell Treatment

IPEC-J2 cells were cultured in DMEM basic (Gibco, Waltham, MA, USA) supplemented with 10% fetal bovine serum (Vigorous Cell Technology Co., Ltd., Shanghai, China) and 1% penicillin–streptomycin mixture. The incubation condition was a humidified incubator at 37 °C with 5% CO_2_. When IPEC-J2 cells reached approximately 80% confluence in 6-well plates, they were assigned to a control group or ETEC-K88 challenge groups. To determine the infection dose, cells were challenged with ETEC-K88 at Multiplicity of Infection (MOI) = 10 or MOI = 100, and samples were challenged with ETEC-K88 and harvested at 12 h (*n* = 6). Afterward, cells were harvested for subsequent index detection, as well as RNA and total protein extraction.

### 2.4. ETEC-K88 Adhesion Assay

To examine the temporal pattern of bacterial adhesion, IPEC-J2 cells were infected with *ETEC-K88*-GFP at MOI 10 in 6-well plates (*n* = 6). Fluorescence images were collected at different post-infection time points, and fluorescence intensity was quantified to estimate the adherent bacterial load. We captured fluorescence images using a Zeiss fluorescence microscope (ZEISS Axio Vert.A1, Baden-Württemberg, Germany), and the fluorescence intensity was analyzed via Image-J software (java 8) (NIH, New York, NY, USA).

### 2.5. Tryptophan Metabolites Measurement

For tryptophan metabolite analysis, IPEC-J2 cells were seeded in 6-well plates and infected with ETEC-K88 at MOI 10 or MOI 100 for 12 h (*n* = 6). After infection, non-adherent bacteria were removed by PBS washing. Cells were lysed and intracellular tryptophan (trp) (CB10334-Pg, COIBO BIO, Shanghai, China), kynurenine (KYN; CB10290-Pg, COIBO BIO, Shanghai, China) and 5-hydroxytryptamine (5-HT; CB10003-Pg, COIBO BIO, Shanghai, China) concentrations were quantified using commercial ELISA kits according to the manufacturer’s instructions.

### 2.6. Tryptophan Supplementation Assay

To test whether L-tryptophan (HY-N0623, MedChemExpress, Shanghai, China) supplementation alleviated *ETEC-K88*-induced epithelial injury, IPEC-J2 cells were assigned to control, *ETEC-K88* and *ETEC-K88* + L-tryptophan groups. Referring to our previous study, the supplemented concentration of L-tryptophan was set at 4 mM [28]. The IPEC-J2 cells were treated with L-tryptophan for 2 h prior to ETEC-K88 infection (*n* = 6). Samples were collected at 8 h post-infection for analysis of transporter-channel genes, aquaporins, tryptophan metabolic enzyme genes, cell-cycle-related genes and signaling-pathway-related genes.

### 2.7. ETEC-K88-Induced Diarrhea Model in Mice

The 4-week-old male C57BL/6J (12 mice) and Balb/c (12 mice) mice were fed a maintenance diet (SL0025, Shulb, Wuhan, China). The mice were maintained in environmentally controlled conditions (23 ± 2 °C, 40–60% humidity, and a 12 h light/dark cycle). After an adaptation period of 1 week, mice were divided into four groups: C57BL/6J control (*n* = 6), C57BL/6J ETEC-K88 (*n* = 6), BALB/c control (*n* = 6) and BALB/c ETEC-K88 (*n* = 6). Intestinal commensal microbiota were depleted using streptomycin sulfate for 2 days before challenge. The administration route was intragastric gavage. Control mice were administered 1 mL PBS, whereas challenged mice were administered *ETEC-K88* (1 mL, 1 × 10^8^ CFU/mL) suspension by gavage for four consecutive days. After challenge, the mice were anesthetized with isoflurane and then sacrificed by cervical dislocation. Then, intestinal tissues were collected, including jejunum, ileum and colon, for gene-expression analysis. And the proximal, middle and distal segments of the jejunum, ileum and colon were collected and fixed in 4% paraformaldehyde for hematoxylin–eosin staining.

### 2.8. RT-qPCR

Total RNA was extracted using TRIzol reagent (TransGen Biotech, Beijing, China). RNA concentration and purity were determined using a NanoDrop One spectrophotometer (Thermo Fisher Scientific, Waltham, MA, USA). Total RNA was reverse transcribed into cDNA using a reverse transcription kit (RR036A, Takara, Dalian, China). RT-qPCR was performed using SYBR qPCR Master Mix (Accurate Biology, Changsha, China) on a Real-Time PCR System (LightCyclerR480II, Roche, Basilea, Switzerland). A melting curve analysis was performed to verify the specificity of amplification. The primers for RT-qPCR are listed in Table 1. The mRNA expressions were calculated using the 2^−ΔΔCt^ method after normalization with the reference gene GAPDH or beta-actin.

### 2.9. Hematoxylin–Eosin (HE) Staining

The proximal, middle and distal segments of the jejunum, ileum and colon were used for HE staining. All procedures were performed in accordance with the manufacturer’s instructions (Biossci, Wuhan, China). After the following procedures: dehydration (100092683, Sinopharm Chemical Reagent Co., Ltd., Shanghai, China), paraffin embedding (10023418, Sinopharm Chemical Reagent Co., Ltd., Shanghai, China), sectioning (RM2016, Leica, Shanghai, China), dewaxing, HE staining (G1005, SERVICEBIO, Wuhan, China) and mounting (10004160, Sinopharm Chemical Reagent Co., Ltd., Shanghai, China), tissue images were captured under a light microscope (Nikon Eclipse E100, Nikon, Tokyo Metropolis, Japan) with a microscopic imaging system (NIKON DS-U3, Nikon, Tokyo Metropolis, Japan) to observe villus height, crypt depth and epithelial integrity. Image J software (NIH, New York, USA) was used for quantitative measurement of villus height and crypt depth.

### 2.10. Statistical Analysis

All experiments were independently repeated at least three times. Statistical analyses were performed using GraphPad Prism 5 (GraphPad Software, San Diego, CA, USA), and differences were assessed by Student’s t-test and one-way analysis of variance (ANOVA). Data are presented as mean ± standard error (SEM). *p* < 0.05, * *p* < 0.01, or different letters indicate statistically significant differences.

## 3. Results

### 3.1. Expression of Gut Barrier and Proliferation-Related Genes in IPEC-J2 Cells Infected with Different Concentrations of ETEC-K88

**Figure 1:**
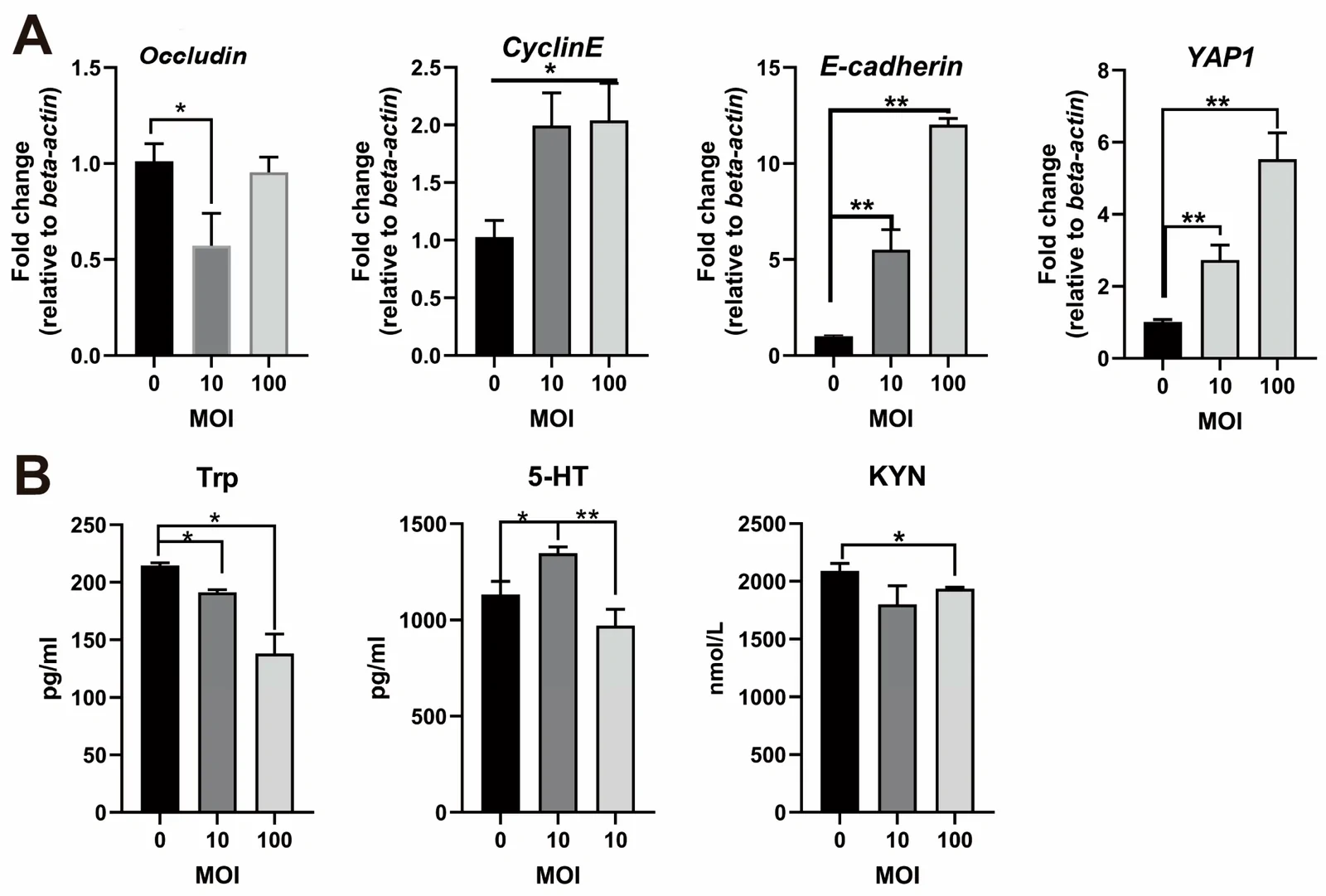
Different effects of MOI on IPEC-J2 cells. (**A**) Fold changes in the expression of *Occludin*, *CyclinE*, *E-cadherin*, and *YAP1* genes relative to the internal reference (*Beta-actin*) under treatments with different MOIs (0, 10, and 100). (**B**) Changes in the concentrations of tryptophan (Trp), serotonin (5-HT), and kynurenine (KYN) under different MOI treatments. Data are presented as mean ± SEM. * *p* < 0.05, ** *p* < 0.01.

Before conducting the time-course infection assay, we compared two multiplicities of infection, MOI 10 and MOI 100, to determine an effective *ETEC-K88* challenge condition in IPEC-J2 cells. At MOI 10, *ETEC-K88* significantly downregulated Occludin expression and increased the mRNA levels of *Cyclin E*, *E-cadherin* and *YAP1*. At MOI 100, *Cyclin E, E-cadherin* and *YAP1* were also significantly increased (Figure 1A). *ETEC-K88* infection also changed intracellular tryptophan metabolites, with marked alterations in tryptophan and 5-hydroxytryptamine levels. Kynurenine was significantly reduced only under the MOI 100 condition (Figure 1B). These results indicated that *ETEC-K88* infection rapidly affected epithelial barrier-, proliferation- and tryptophan-metabolism-related indices in IPEC-J2 cells.

### 3.2. Temporal Distribution of ETEC-K88 Adhesion to IPEC-J2 Cells

**Figure 2:**
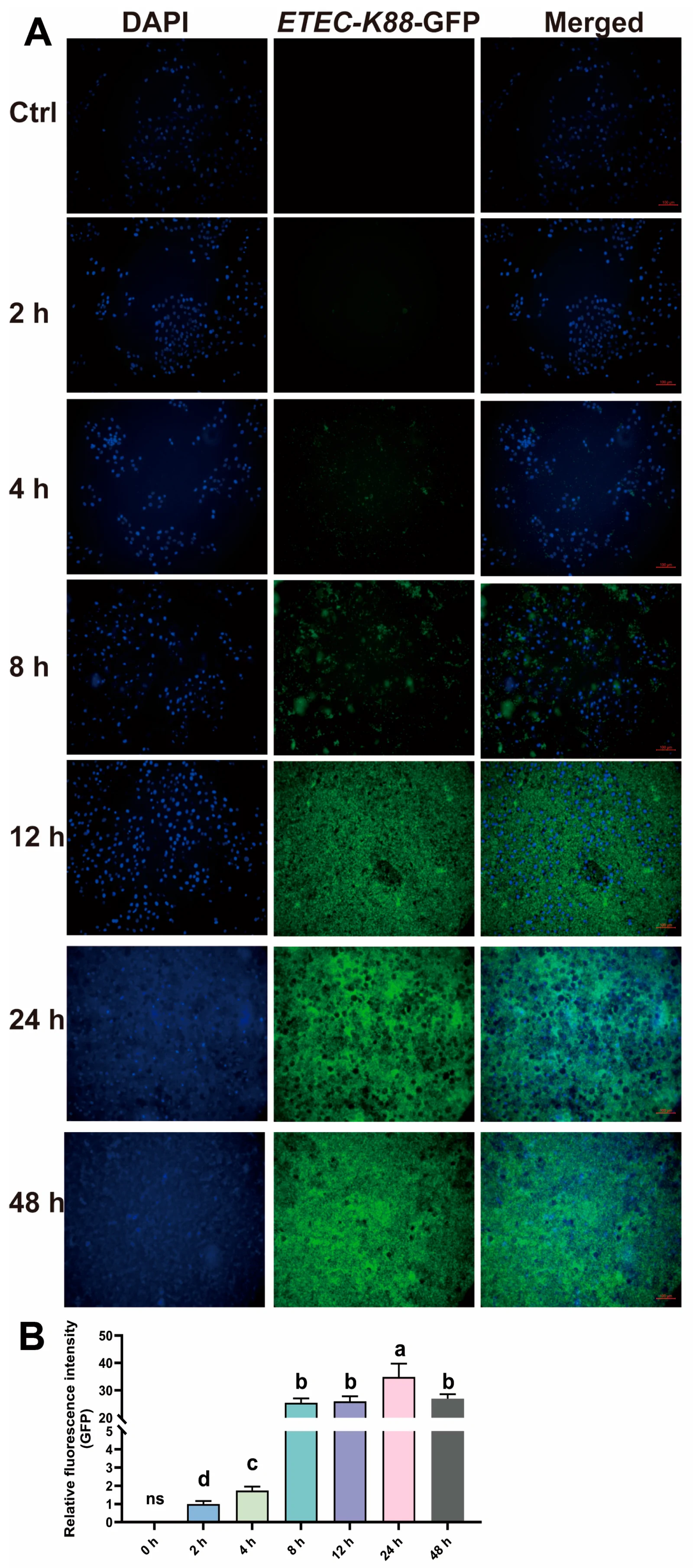
Fluorescence observation and quantitative analysis of *ETEC-K88*-GFP adhesion/invasion to cells at different time points. (**A**) Representative fluorescence microscopy images showing the interaction between *ETEC-K88*-GFP and cells at different time points (0, 2, 4, 8, 12, 24, and 48 h). Cell nuclei were stained with DAPI (blue), and *ETEC-K88* was labeled with GFP (green). (**B**) Quantitative analysis of relative GFP fluorescence intensity at different time points. Data are presented as mean ± SEM. Different lowercase letters indicate significant differences between groups (*p* < 0.05).

GFP-labeled ETEC-K88 was used to characterize the temporal distribution of bacterial adhesion to IPEC-J2 cells. During the first 4 h post-infection, only a small number of bacteria adhered to the cells. The adherent bacterial load increased rapidly after 4 h and reached its highest level at 24 h. Between 8 h and 12 h, adhesion entered a plateau phase, whereas a slight decrease was observed after 24 h (Figure 2A). These findings identified 4 h post-infection as a transition point at which bacterial adhesion began to increase markedly (Figure 2B).

### 3.3. Expression of Genes Related to Tryptophan Metabolic Enzymes and Intestinal Barrier Function in IPEC-J2 Cells with ETEC-K88 Infection

**Figure 3:**
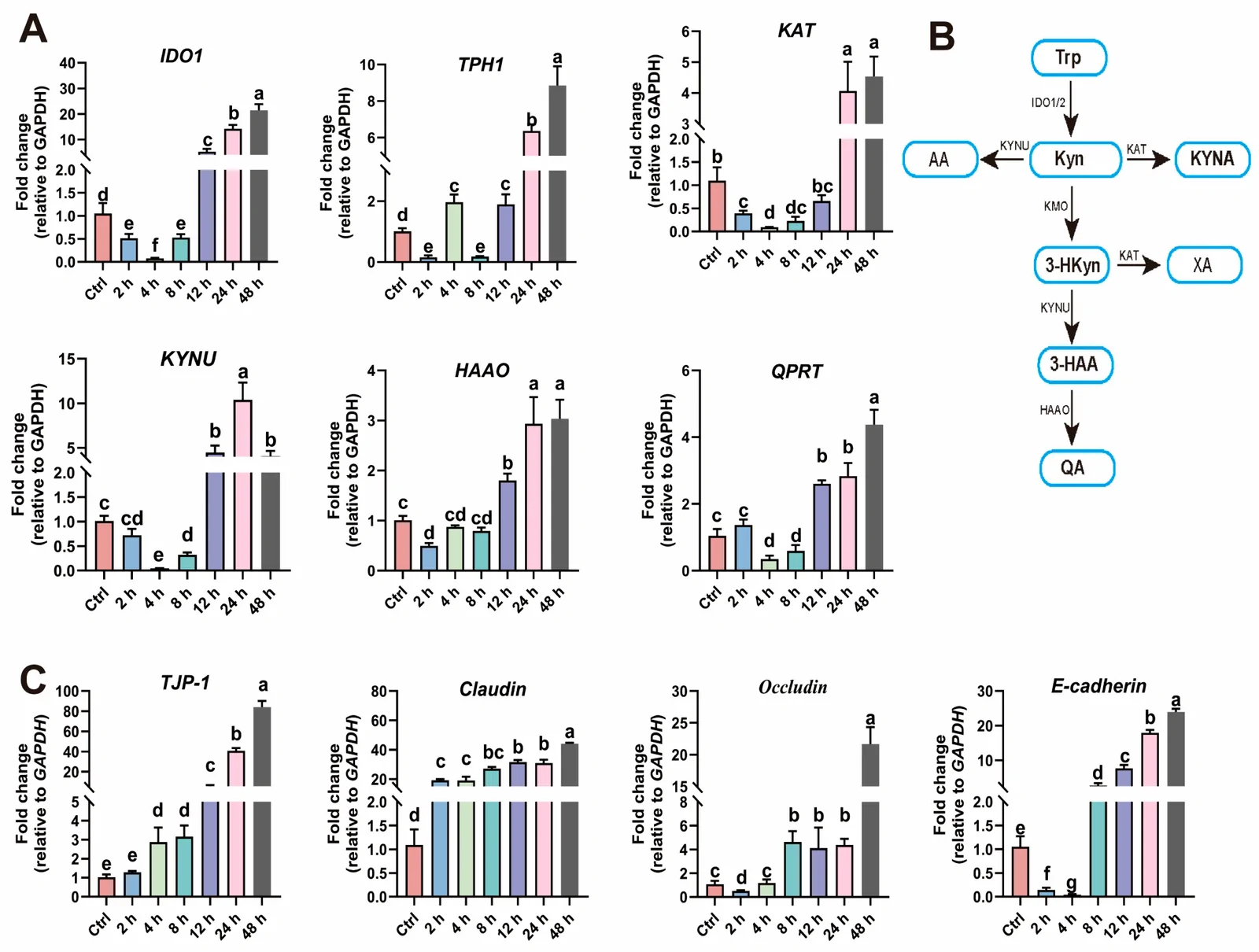
Expression analysis of tryptophan metabolic pathway and barrier-related genes at different time points. (**A**) Fold changes of key enzyme genes in tryptophan metabolism (*IDO1*, *TPH1*, *KAT*, *KYNU*, *HAAO*, *QPRT*) relative to *GAPDH* at different time points (Ctrl, 2, 4, 8, 12, 24, and 48 h). (**B**) Schematic diagram of the tryptophan metabolic pathway, showing the main metabolites (Trp, Kyn, KYNA, 3-HKyn, XA, 3-HAA, QA) and their corresponding catalyzing enzymes. (**C**) Expression of tight junction and adhesion-related genes (*TJP-1*, *Claudin*, *Occludin*, *E-cadherin*) at different time points. Data are presented as mean ± SEM. Different lowercase letters indicate significant differences between groups (*p* < 0.05).

Next, we quantified the expression of genes encoding tryptophan metabolic enzymes and epithelial barrier components during ETEC-K88 infection. Kynurenine-pathway genes, including *IDO1*, *KAT*, *KYNU* and *QPRT*, were significantly downregulated during the early 0–4 h infection window and were subsequently upregulated from 8 h to 48 h. *TPH1* showed a similar dynamic pattern, with decreased expression during early infection, followed by increased expression after 8 h (Figure 3A,B). Barrier-related genes displayed distinct temporal profiles. *TJP-1* and *Claudin 1* were significantly upregulated from 2 h to 48 h, whereas *Occludin* and *E-cadherin* were downregulated during early infection and increased after 4 h (Figure 3C). These results suggest that *ETEC-K88* infection first suppresses several tryptophan metabolic and junctional markers before triggering a later compensatory transcriptional response.

### 3.4. Expression of Proliferation and Apoptosis-Related Genes in IPEC-J2 Cells with ETEC-K88 Infection

**Figure 4:**
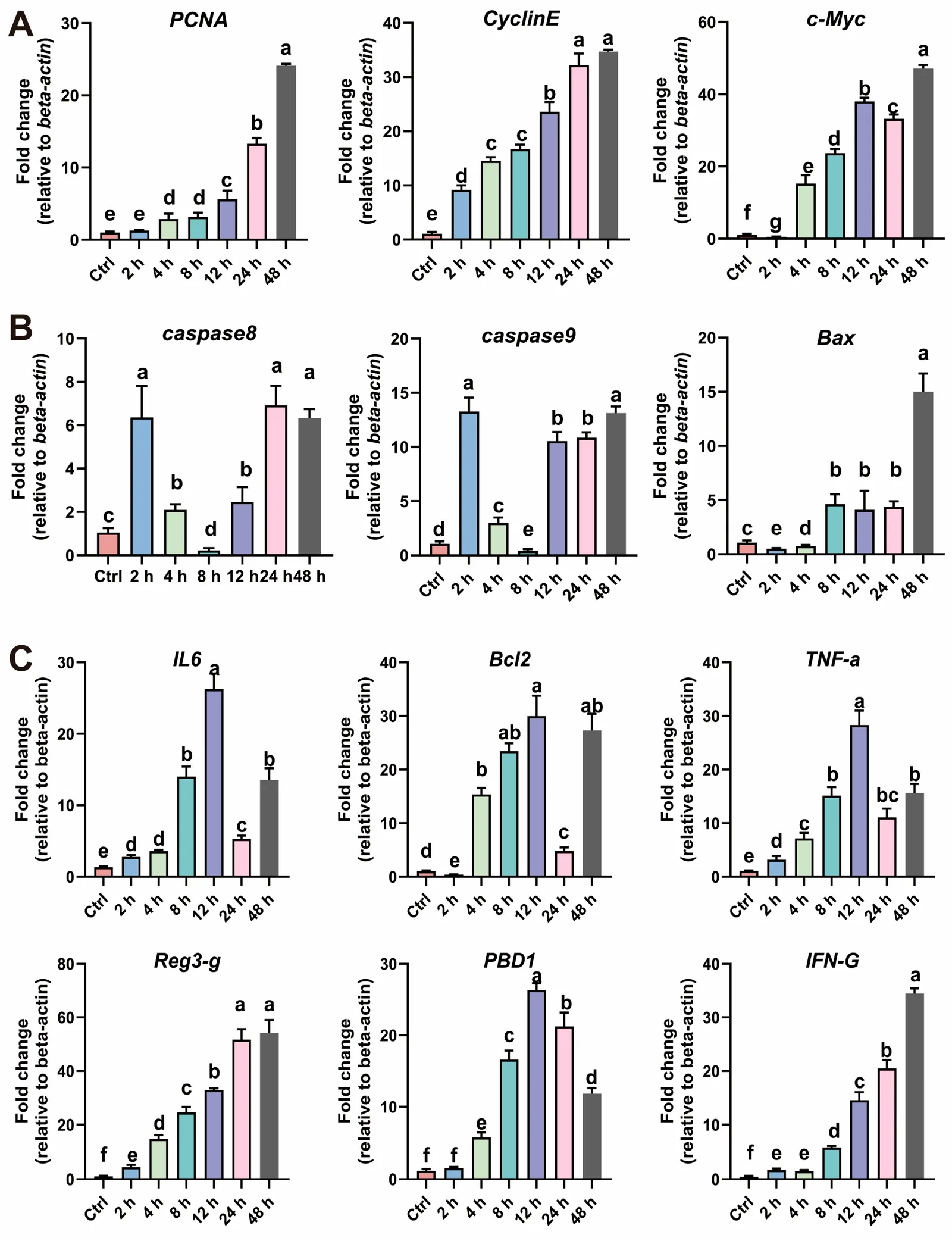
Expression changes of genes related to proliferation, apoptosis, and immunity at different time points. (**A**–**C**) Expressions of cell proliferation-related genes (*PCNA*, *CyclinE*, *c-Myc*) (**A**), apoptosis (*caspase8*, *caspase9*, *Bax*) (**B**) and immune and inflammation-related genes (*IL6*, *Bcl2*, *TNF-a*, *Reg3-g*, *PBD1*, *IFN-G*) (**C**) at different time points. Data are presented as mean ± error bars. Different lowercase letters indicate significant differences between groups (*p* < 0.05).

Similarly, we quantified the expression of cell cycle and apoptosis-related genes following *ETEC-K88* infection. Cell cycle-related genes (*PCNA*, *Cyclin E*, *c-Myc*) shared similar expression profiles. All three genes were significantly upregulated from 0 h to 48 h with *ETEC-K88* infection (Figure 4A). Genes associated with cell apoptosis displayed a dynamic pattern of initial increase, subsequent decrease and final re-increase. The overall expression of apoptotic genes (*Caspase8*, *Caspase9*) was markedly elevated at 0–2 h post-infection, significantly reduced during 2–8 h, and remarkably upregulated again from 8 h to 48 h. Specifically, the pro-apoptotic gene *Bax* was significantly downregulated at 0–4 h, followed by a notable upregulation during 8–48 h (Figure 4B). Finally, we determined the mRNA levels of several inflammatory cytokines and antimicrobial peptide genes. The expression of *IL6, Bcl2* and *TNF-α* was significantly increased at 2–12 h, followed by a remarkable reduction at 12–24 h and another significant upregulation at 24–48 h. *Reg3γ, IFN-γ* and *PBD-1* were all significantly upregulated within 2–12 h. Thereafter, porcine *β-defensin 1* (*PBD-1*) expression decreased, whereas *Reg3γ* and *IFN-γ* remained significantly upregulated (Figure 4C). These coordinated responses indicate that ETEC-K88 infection altered epithelial turnover and immune-defense programs in a time-dependent manner.

### 3.5. Expression of Aquaporin and Transporter Channel-Related Genes in IPEC-J2 Cells with ETEC-K88 Infection

**Figure 5:**
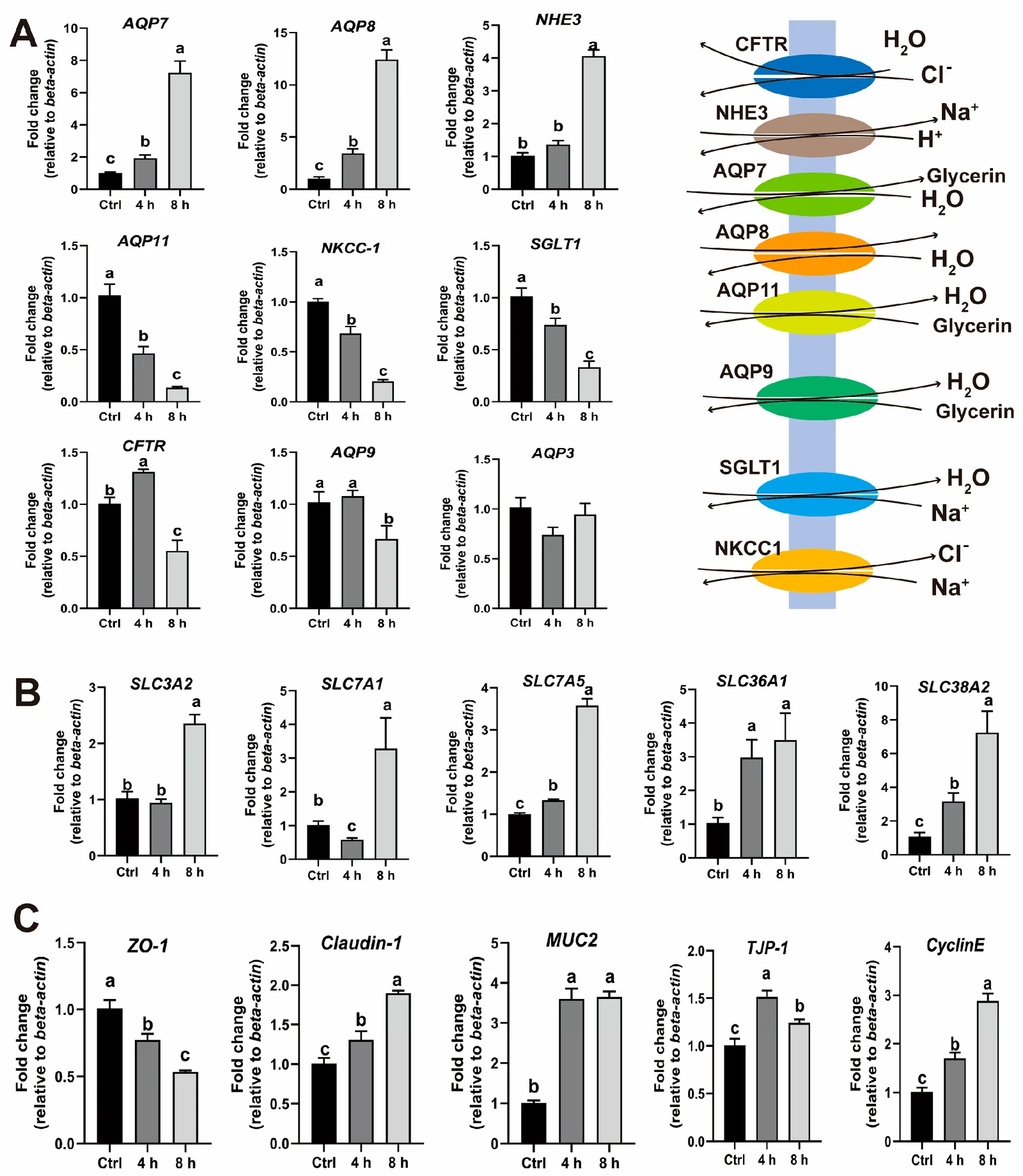
Expression of transport channels, solute carriers, and barrier function-related genes in IPEC-J2 following 4–8 h of ETEC-K88 infection. (**A**) The expressions of aquaporin, ion transport, and electrolyte carrier-related genes (*AQP7*, *AQP8*, *NHE3*, *AQP11*, *NKCC-1*, *SGLT1*, *CFTR*, *AQP9*, *AQP3*) at different time points, along with a schematic diagram showing the localization and transported substances of relevant transporters. (**B**,**C**) Relative expression changes of amino acid transport-related genes (*SLC3A2*, *SLC7A1*, *SLC7A5*, *SLC36A1*, *SLC38A2*) (**B**), intestinal barrier genes (ZO-1, *Claudin-1*, *MUC2*, *TJP-1*) and a proliferation-related gene (*CyclinE*) at different time points (**C**). Data are presented as mean ± SEM. Different lowercase letters indicate significant differences between groups (*p* < 0.05).

Because the 4–8 h period appeared to be a critical transition window, we further examined aquaporin, solute carrier and epithelial barrier genes at these time points. The results revealed that *AQP7, AQP8* and *NHE3* were significantly upregulated from 4 h to 8 h. In contrast, *AQP11*, *NKCC1*, *SGLT1*, *CFTR* and *AQP9* exhibited marked downregulation during the same period (Figure 5A). For solute carrier family (SLC) transporters, the expression levels of *SLC3A2*, *SLC7A1*, *SLC7A5*, *SLC36A1* and *SLC38A2* were markedly increased at 4–8 h post-infection. Notably, *SLC7A1* exhibited a time-dependent change, which was significantly downregulated within 0–4 h after bacterial challenge (Figure 5B). Lastly, we quantified the expression of intestinal barrier-related genes at 4 h and 8 h post-infection. Compared with the control group, the expression levels of *Claudin-1*, *MUC2, TJP-1* and *Cyclin E* were significantly upregulated at both 4 h and 8 h, whereas *ZO-1* was markedly downregulated at the two time points (Figure 5C). Together, these data indicate that the early adhesion transition coincided with broad remodeling of water transport, solute transport and epithelial barrier-associated transcription.

### 3.6. Effect of Tryptophan Supplementation on the Alleviation of ETEC-K88-Induced Damage in IPEC-J2 Cells

**Figure 6:**
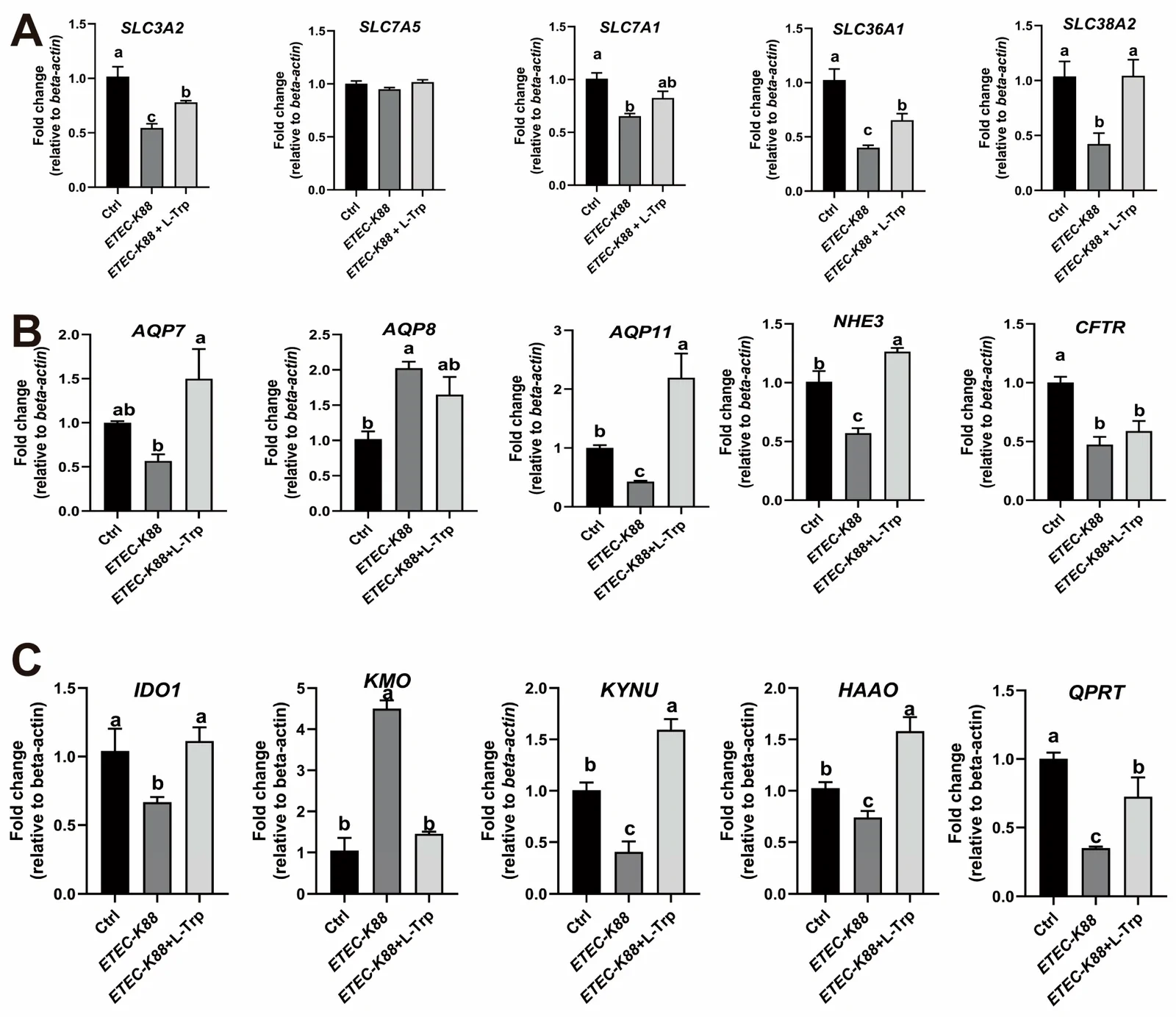
Effects of L-tryptophan (L-Trp) supplementation in IPEC-J2 with *ETEC-K88* infection. (**A**–**C**) The changes in amino acid transport-related genes (*SLC3A2*, *SLC7A5*, *SLC7A1*, *SLC36A1*, *SLC38A2*) (**A**), aquaporin and ion transport genes (*AQP7*, *AQP8*, *AQP11*, *NHE3*, *CFTR*) (**B**) and key tryptophan metabolism enzyme genes (*IDO1*, *KMO*, *KYNU*, *HAAO*, *QPRT*) (**C**) in *ETEC-K88*+L-Trp compared with the *ETEC-K88* group. Data are presented as mean ± SEM. Different lowercase letters indicate significant differences between groups (*p* < 0.05).

During *ETEC-K88* infection, the concentration of Trp was significantly decreased. Accordingly, we investigated whether exogenous tryptophan supplementation could alleviate the cellular damage induced by *ETEC-K88* infection in IPEC-J2 cells. The results demonstrated that L-tryptophan addition remarkably restored the abnormal expression of transporter channel-related genes and aquaporin genes triggered by *ETEC-K88* challenge (Figure 6A). Compared with the control group, the expression of *AQP7, AQP11* and *NHE3* was markedly downregulated in the single *ETEC-K88* infection group, whereas these genes were significantly upregulated in the *ETEC-K88* + L-Trp group relative to the *ETEC-K88* group alone (Figure 6B). A similar regulatory pattern was observed in tryptophan metabolic enzyme genes. The expression levels of *IDO1*, *KYNU*, *HAAO* and *QPRT* were significantly reduced in the *ETEC-K88* group compared to the control group, and their expression exhibited obvious elevation in the *ETEC-K88* + L-Trp group compared with the *ETEC-K88* group. In contrast, *KMO* exhibited an expression pattern opposite to that of the other tryptophan metabolic enzyme genes across the three groups (Figure 6C). Lastly, we further quantified the expression of cell cycle-related genes. Tryptophan supplementation effectively rescued the downregulation of cell cycle-related genes induced by *ETEC-K88* infection in IPEC-J2 cells. Further pathway analysis revealed that tryptophan treatment exerted no significant effects on the expression of genes involved in the WNT/β-catenin signaling pathway (*β-catenin*, *c-Myc*, *Cyclin E*, *Cyclin D1*, *PCNA*), whereas it significantly impacted the expression of Hippo signaling pathway-related genes (*YAP1*, *MST1*, *Cyclin E*, *Cyclin D1*, *PCNA*) (Figure S1).

### 3.7. Strain- and Segment-Specific Intestinal Gene Expression in Mice with ETEC-K88 Infection

**Figure 7:**
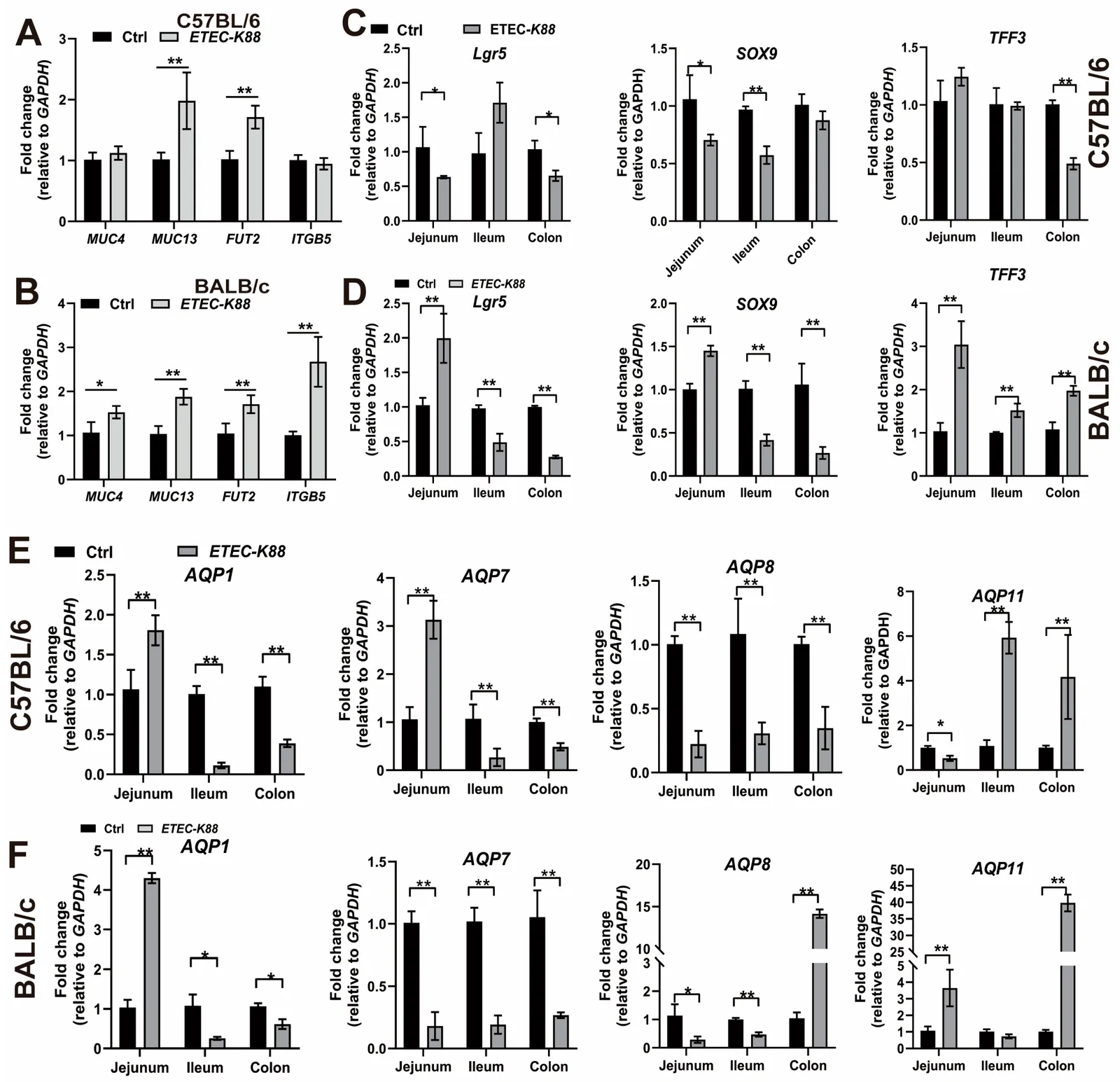
Effects of ETEC-K88 infection in different intestinal segments of BALB/c and C57BL/6 mice. (**A**,**B**) Effect of ETEC-K88 infection on the relative expression of ETEC-K88 fimbrial receptor genes (*MUC4*, *MUC13*, *FUT2*, *ITGB5*) in the colon of C57BL/6 (**A**) and BALB/c (**B**) mice. (**C**,**D**) Effect of ETEC-K88 infection on the relative expression of intestinal stem cell and goblet cell-related genes (*Lgr5*, *SOX9*, *TFF3*) in the jejunum, ileum, and colon of C57BL/6 (**C**) and BALB/c (**D**) mice. (**E**,**F**) Effect of ETEC-K88 infection on the relative expression of aquaporin genes (*AQP1*, *AQP7*, *AQP8*, *AQP11*) in the colon of C57BL/6 (**E**) and BALB/c (**F**) mice. Data are presented as mean ± error bars. Compared with the control group, * *p* < 0.05, ** *p* < 0.01.

To evaluate the infection efficiency of ETEC K88 in mice, we challenged C57BL/6J and BALB/c mice with *ETEC-K88*. Although overt diarrhea was not induced, *ETEC-K88* infection significantly altered the colon length of mice (Figure S2A,C). In addition, compared with the control group, no significant difference was detected in fecal scores (Figure S2B,D). However, the ETEC-K88 fimbrial receptor genes were both significantly upregulated in C57BL/6J and BALB/c mice, including *MUC4*, *MUC13*, *FUT2*, and *ITGB5* in BALB/c (Figure 7B) and *MUC13* and *FUT2* in C57BL/6J (Figure 7A). *ETEC-K88* significantly regulated intestinal stem cell markers, the goblet cell marker *TFF3* and aquaporin genes in the jejunum, ileum and colon. In C57BL/6J mice, *Lgr5* was downregulated in the jejunum, upregulated in the ileum and downregulated in the colon, whereas *SOX9* was downregulated in the jejunum and ileum. *TFF3* was significantly downregulated only in the colon (Figure 7C). Aquaporin genes exhibited segment-specific regulation, with increased expression of *AQP1*, *AQP7* and *AQP11* in the jejunum but reduced expression of *AQP1* and *AQP7* in the ileum and colon (Figure 7E).

BALB/c mice exhibited a distinct response pattern. *Lgr5* and *SOX9* were significantly upregulated in the jejunum but downregulated in the ileum and colon. In contrast to C57BL/6J mice, *TFF3* was significantly upregulated in the jejunum, ileum and colon (Figure 7D). Aquaporin responses were also strain- and segment-dependent: *AQP1* was upregulated in the jejunum but inhibited in the ileum and colon, *AQP7* was downregulated across all three intestinal segments, *AQP8* was downregulated in the ileum but upregulated in the colon, and *AQP11* was upregulated in the ileum and colon (Figure 7F). These results demonstrate that *ETEC-K88*-induced intestinal molecular responses differ by host genetic background and intestinal location.

### 3.8. Intestinal Histological Traits in Two Different Mouse Strains Following ETEC-K88 Infection

**Figure 8:**
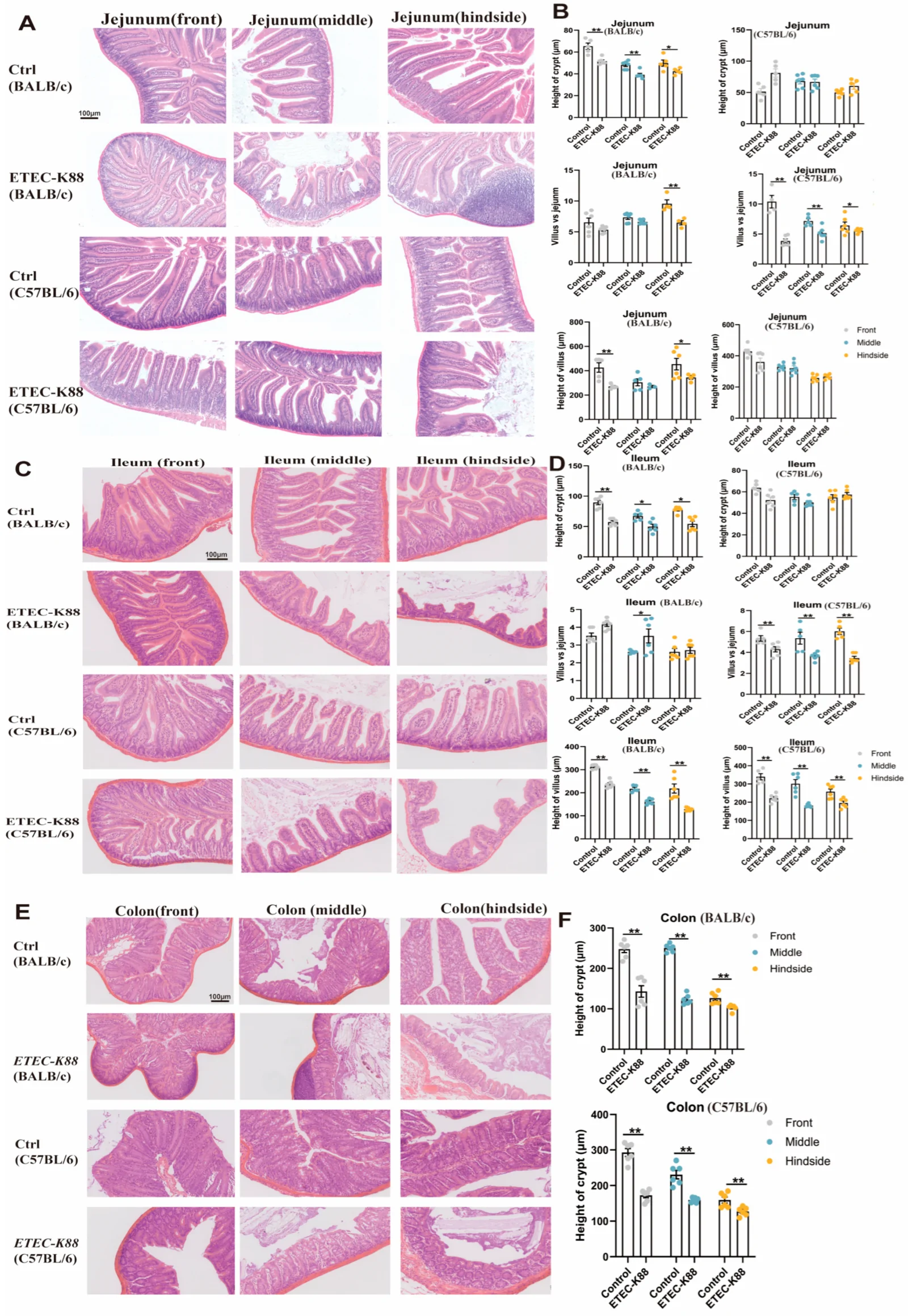
Effects of ETEC-K88 infection on the histomorphology of different intestinal segments of BALB/c and C57BL/6 mice. (**A**–**F**) Representative H&E staining images and quantitative statistical analysis of crypt/villus height of the front, middle, and hindside segments of the jejunum (**A**,**B**), ileum (**C**,**D**), and colon (**E**,**F**), respectively (for both mouse strains). Data are presented as mean ± SEM. Compared with the control group, * *p* < 0.05, ** *p* < 0.01.

Hematoxylin–eosin staining demonstrated that control mice possessed intact villi, orderly epithelial structures and normal crypt morphology in the jejunum, ileum and colon. In contrast, *ETEC-K88*-challenged mice displayed intestinal mucosal injury, including shortened and disorganized villi, epithelial shedding in the jejunum and ileum, disrupted crypt architecture and thinner colonic mucosa. These histopathological alterations were consistently observed in both BALB/c and C57BL/6 mouse strains (Figure 8A,C,E).

Further quantitative analysis of the jejunal villi and crypts indicated that, compared with their respective controls, the *ETEC-K88* groups of both mouse strains exhibited significant decreases in most parameters across the front, middle, and hind segments; however, notable exceptions were found in the C57BL/6 strain, where the crypt depth in the front segment and the villus-to-crypt (V/C) ratio in the middle segment were significantly increased (Figure 8B). In the ileum, villus height was significantly reduced across all three segments in both BALB/c and C57BL/6 mice following *ETEC-K88* infection. Regarding strain-specific changes in the ileum, the BALB/c mice exhibited significantly decreased crypt depth in the front and hind segments, but a significantly increased V/C ratio in the middle segment. Conversely, the C57BL/6 mice exhibited significant reductions in both the V/C ratio and crypt depth in the middle and hind segments (Figure 8D).

Regarding the colon, the crypt height in the *ETEC-K88* groups was significantly lower than that in the corresponding control groups for both BALB/c and C57BL/6 mice in the front and middle segments. And the distance from the base of the crypt to the submucosa was increased in *ETEC-K88* groups than in control groups (Figure 8F).

## 4. Discussion

In this study, we provide a time-resolved map of ETEC-K88-induced epithelial responses rather than relying on a single-endpoint infection model. Our results revealed that 4–8 h represents a critical window, during which bacterial adhesion increased markedly, accompanied by significant changes in the expression of genes related to immunity, barrier function, and aquaporins. These findings were consistent with the expression patterns of the crucial inflammatory molecules (IL-8 and MLCK pathways and TNFα and IL1α) observed in previous studies of ETEC infection in IPEC-J2 cells [29,30]. A 2 h observation window was adopted in previous experiments to assess the effects of ZnO on *ETEC-K88* adhesion to IPEC J2 cells [31,32]. Based on our results, substantial bacterial adhesion begins to occur at four hours post-infection. However, the observation time points used in previous studies on barrier integrity, proliferation, inflammation, and antioxidant responses in IPEC-J2 cells are not fully consistent with our findings [33,34,35]. For instance, LPS induced apoptosis at 24 h [36], combined DON and LPS induced barrier damage at 48 h [37,38], and rutin treatment modulated proliferation and barrier function at 24 h [39]. These findings may result from oxidative stress induced by *ETEC-K88* in IPEC-J2, reflecting an early-onset immune response or transient functions. Therefore, our findings aid in selecting appropriate time points to evaluate the expression of diverse functional genes.

Additionally, we discuss aquaporins, ion transporters, and SLC transporters as components of an epithelial transport program potentially associated with ETEC-related fluid imbalance. Likewise, within the time window of 4 to 8 h after infection, the expression of genes related to aquaporins and transport proteins was markedly decreased in ETEC-K88-infected IPEC J2 cells. Aquaporins and ion transporters play critical roles in piglet diarrhea and represent the ultimate targets of enterotoxins released by ETEC-K88 [40,41]. The most vital phenotype of diarrhea is excessive moisture in feces, which arises from the disruption of intestinal aquaporins and ion channels. In previous studies, researchers have reported downregulated expression of AQP4 and AQP8 in the diarrheic colon [42,43,44]. Morphine induces severe constipation by elevating the expression level of AQP3 within the colon [45]. Based on our results, aquaporin expression was significantly reduced by *ETEC-K88* infection, consistent with the results of previous studies. Based on these findings, researchers conducting subsequent aquaporin-related research should consider the 4–8 h post-infection time for sampling and investigation.

In addition, tryptophan metabolism dysregulation is observed in patients with diarrhea [20,46], and this phenomenon is similarly present in piglet diarrhea induced by ETEC-K88 infection [23,47]. The results of a number of studies have demonstrated that supplementation with tryptophan or its metabolites alleviates barrier damage and inflammatory responses [47,48,49]. Similarly, we also detected significant alterations in tryptophan metabolism during ETEC-K88 infection of IPEC-J2 cells and subsequently supplemented the cells with tryptophan. Within the 4–8 h infection window, we observed that tryptophan supplementation attenuated intestinal barrier disruption, inflammation, and the dysregulation of aquaporins and transporters. These results could provide a basis for the regulation of intestinal health via nutritional intervention with tryptophan.

IPEC-J2 cells are a monolayer of small intestinal epithelial cells; they are characterized by inherent limitations, and piglet models are constrained by housing requirements and experimental duration, making them unsuitable for all experimental studies. We therefore aimed to establish a mouse diarrhea model induced by ETEC-K88 infection. However, infection with *ETEC-K88* in both C57BL/6 and BALB/c mice did not produce an overt diarrheal phenotype, although significant intestinal lesions were observed, combined with marked changes in the expression of genes encoding aquaporins, stem cell markers, and goblet cell markers. *Campylobacter jejuni* [50], enteropathogenic *E. coli* (EPEC) [51,52], and *Escherichia coli* O157:H7 [53,54] have all been used to successfully establish mouse diarrhea models and exhibit changes in aquaporins and intestinal epithelial cells similar to those associated with *ETEC-K88* infection. Moreover, the *EEC-K88* fimbria receptor genes were expressed in both C57BL/6 and BALB/c mice. The *EEC-K88* fimbria receptors are key proteins that recognize and mediate the adhesion of *ETEC-K88* [3,55]. Porcine-derived *ETEC-K88* is primarily adapted to pigs as a neutral host and exhibits limited susceptibility in mice, which may explain the absence of diarrheal phenotypes. The failure of *ETEC-K88* to trigger diarrhea in mice may be attributed to pigs being its natural host. Despite antibiotic pretreatment, the bacterium exhibits poor effective colonization within the mouse intestine. Another possible explanation is that a higher bacterial concentration or a prolonged infection duration may be required for *ETEC-K88* challenge in the mouse model. This hypothesis awaits further investigation.

## 5. Conclusions

Overall, through a time-course analysis, we have systematically characterized the dynamic expression pattern of genes related to intestinal barrier function, tryptophan metabolism and aquaporins in IPEC J2 cells upon *ETEC-K88* infection at different time points. We show that 4 to 8 h after *ETEC-K88* infection is the most profound time point for inducing pathological phenotypes in IPEC-J2 cells. Furthermore, tryptophan addition is able to alleviate ETEC-K88 infection-induced intestinal cell dysfunction in IPEC-J2 cells. Finally, by further establishing the *ETEC-K88*-infected mouse models, we have revealed strain- and segment-specific intestinal morphological abnormalities between C57BL/6J and BALB/c mice after infection. These findings provide a framework for selecting effective infection time points and testing nutritional interventions, particularly tryptophan-based strategies, in pig production.

## Figures and Tables

**Table 1:** Primer sequences of target genes.

Models	Gene	Primer Sequences (5′ → 3′)
**IPEC-J2**	***c-Myc***	F: TCCACGCACCAGCACAATTA
		R: TCGTTTCTCCTCTGGCGTTC
	***Cyclin D1***	F: CCCTCCGTGTCCTACTTCAA
		R: CCTCGCACTTCTGCTCCTC
	***Cyclin E***	F: CTCGCCACTGCCTATACTGA
		R: GGTGCCGCTGCATAAGGT
	***PCNA***	F: TACGCTAAGGGCAGAAGATAATG
		R: CTGAGATCTCGGCATATACGTG
	***E-cadherin***	F: TTCAACCCAACCTCGTACCA
		R: CGCCTTCATTGGTTACTGGG
	***Caspase-8***	F: AGGAATCAGGACGTGTGAAG
		R: GAGGATGTAGTTGTAACCGTG
	***Caspase-9***	F: ACATCCAGGTGGTCCAG
		R: CTCTCACAGTCGATATTG
	***Bax***	F: TTTCTGACGGCAACTTCAACTGGG
		R: TGTCCAGCCCATGATGGTTCTGAT
	***Bcl-2***	F: TTCTTTGAGTTCGGTGGGG
		R: CCAGGAGAAATCAAATAGAGGC
	***IDO1***	F: AACGCTCAGCATTGACG
		R: GCAGGACCTTGCGGATA
	***KAT***	F: GGCAAGGTGTTCTCCAAG
		R: GCTGGTACTGGTCGTAGA
	***KYNU***	F: ACCTGCCATCACAAAAGCTGG
		R: TAGGAGCACCAGCAGGCAAA
	***HAAO***	F: AGCAGCCACAGGGTATGTCC
		R: CTTGGCAGCAGCACACGTAG
	***QPRT***	F: GGAGCGGGTGGCCCTTAATA
		R: GGTCGTACCTGTGGGAGGTG
	***TPH1***	F: TGGATCTGAACTGGATGCTG
		R: CGGTTCCCCAGGTCTTAATC
	***TNF-α***	F: ATCGGCCCCCAGAAGGA
		R: GATGGCAGAGAGGAGGT
	***IFN-γ***	F: TCAAAGATAACCAGGCCATTCA
		R: CAGTTTCCCAGAGCTACCATTTA
	***IL-6***	F: TGCTTCTGGTGATGGCTACTG
		R: CCGGAGAGGTGAAGAGCATTT
	***PBD-1***	F: TACCTGTGCCAGGTCTACTA
		R: TTTCATCTTTGGAGCACACT
	***RegIII-γ***	F: CCATCTTCACGTAGCAGC
		R: CAAGATGTCCTGAGGGC
	***AQP1***	F: GCCAGCGAGTTCAAGAAGAAG
		R: ACCGATGCTGATGAAGATGAAG
	***AQP7***	F: TTCTCAGAGGGAAAGCTGACAGT
		R: GTTGAGGAAGATAGGTGGCAAAA
	***AQP8***	F: CCATTCTCCATCGGCTTCTC
		R: AGCAGGTCCAAAGGCTCGT
	***AQP11***	F: GCCATCTGCTCCTTTATCTTCC
		R: AAATACCGCTCCTGTTAGACTTCCT
	***β-actin***	F: AGAGCAAGAGAGGCATCCTG
		R: CACGCAGCTCGTTGTAGAAG
	***GAPDH***	F: GGTCGGAGTGAACGGATTTG
		R: ACTGTGCCGTGGAATTTGC
**Mice**	***Lgr5***	F: TGA CTT TGA GGA AGA CCT GAA G
		R: GGA TCA GCC AGC TAC CAA ATA
	***Sox9***	F: CTCGCATACCTCCCTTCC
		R: TTCCAGCAGTCACTAGGC
	***Gapdh***	F: TGC GAC TTC AAC AGC AAC TC
		R: ATG TAG GCC ATG AGG TCC AC
	***TFF3***	F: GTC ACA TCG GAG CAG TGT AA
		R: GTC TCC TGC AGA GGT TTG AA
	***AQP7***	F: ATGGCCCCCAGGTCTGTGCT
		R: CCAACAGGTCTCCGCCTGCA
	***AQP8***	F: AGCATGGCTGGCAGATGCCG
		R: AGGCAGCCCCAATCAGCCCT
	***AQP11***	F: ATGTCCGCGCTACTGGGGCT
		R: GGAGCTCGTGGGTGCAGCAG
	***AQP1***	F: CTGCGGTCACACTGGGGCTC
		R: GCCAGCAGGTGTCCAAGGGC
	***MUC4***	F: TGTGTCCCCATGTAGTGAGG
		R: CCCGTAGAATGCCCCAAGTTT
	***MUC13***	F: GGACCCAGGCAAATGACAATA
		R: CCCTGCTTTCCTACCAACTAAC
	***FUT2***	F: CAGGATGAACGGTCGGCTTGC
		R: TTCTGGCTGTGTCGCTGTGTAAC
	***ITGB5***	F: GTCTGCTAATCCACCCAAAATG
		R: TCTCTATCTCACCTCCACAGC
	***β-actin***	F: GAGACCTTCAACACCCCAGC
		R: GGAGAGCATAGCCCTCGTAGAT
	***GAPDH***	F: TGCCCAGAACATCATCCCT
		R:GGTCCTCAGTGTAGCCCAAGA

## Data Availability

Data supporting the findings of this study can be obtained from the corresponding author.

## Supplementary Materials

The following supporting information can be downloaded at: https://www.mdpi.com/article/10.3390/ani16172632/s1. Figure S1. Effect of L-Trp on proliferation-related genes in ETEC-K88-infected IPEC-J2 cells; Figure S2. Effects of ETEC-K88 infection on intestinal morphology and fecal traits in different mouse strains.

## Abbreviations

IPEC-J2	Intestinal Porcine Epithelial Cell line-J2
ETEC-K88	Enterotoxigenic Escherichia coli K88
GFP	Green fluorescent protein
MOI	Multiplicity of Infection
DMEM	Dulbecco’s Modified Eagle Medium
Trp	Tryptophan
KYN	Kynurenine
5-HT	5-Hydroxytryptamine
IDO1	Indoleamine 2,3-dioxygenase 1
KAT	Kynurenine aminotransferase
KYNU	Kynureninase
QPRT	Quinolinate phosphoribosyltransferase
TPH1	Tryptophan hydroxylase 1
E-cadherin	Epithelial cadherin
ZO1	Zonula occludens protein 1
TJP-1	Tight junction protein 1
Claudin 1	Claudin 1
PCNA	Proliferating cell nuclear antigen
c-Myc	MYC Proto-Oncogene
Bcl2	B-cell lymphoma 2
IL6	Interleukin-6
TNF-α	Tumor necrosis factor-alpha
IFN-γ	Interferon-gamma
Reg3γ	Regenerating islet-derived protein 3-gamma
PBD-1	Porcine beta-defensin 1
MUC2	Mucin-2
Lgr5	Leucine-rich repeat-containing G-protein coupled receptor 5
SOX9	SRY-box transcription factor 9
TFF3	Trefoil factor 3
AQP1	Aquaporin 1
AQP7	Aquaporin 7
AQP8	Aquaporin 8
AQP9	Aquaporin 9
AQP11	Aquaporin 11
NHE3	Sodium/hydrogen exchanger 3
NKCC1	Na^+^-K^+^-Cl- cotransporter 1
SGLT1	Sodium/glucose cotransporter 1
CFTR	Cystic fibrosis transmembrane conductance regulator
SLC3A2	Solute carrier family 3 member 2
SLC7A1	Solute carrier family 7 member 1
SLC7A5	Solute carrier family 7 member 5
SLC36A1	Solute carrier family 36 member 1
SLC38A2	Solute carrier family 38 member 2
YAP1	Yes-associated protein 1
MST1	Macrophage stimulating 1
β-catenin	Beta-catenin
V/C	Villus-to-crypt
