# Supplementary material for: Time-Resolved Epithelial Responses to ETEC-K88 Reveal Tryptophan-Linked Protection in IPEC-J2 Cells and Strain-Specific Intestinal Injury in Mice
Source: Animals (Basel). 2026 Aug 22;16(17):2632. doi: 10.3390/ani16172632 (PMC13564277; doi:10.3390/ani16172632)
Supplement: Supplementary file 1 [file animals-16-02632-s001.zip › animals-4512547-supplementary.pdf]

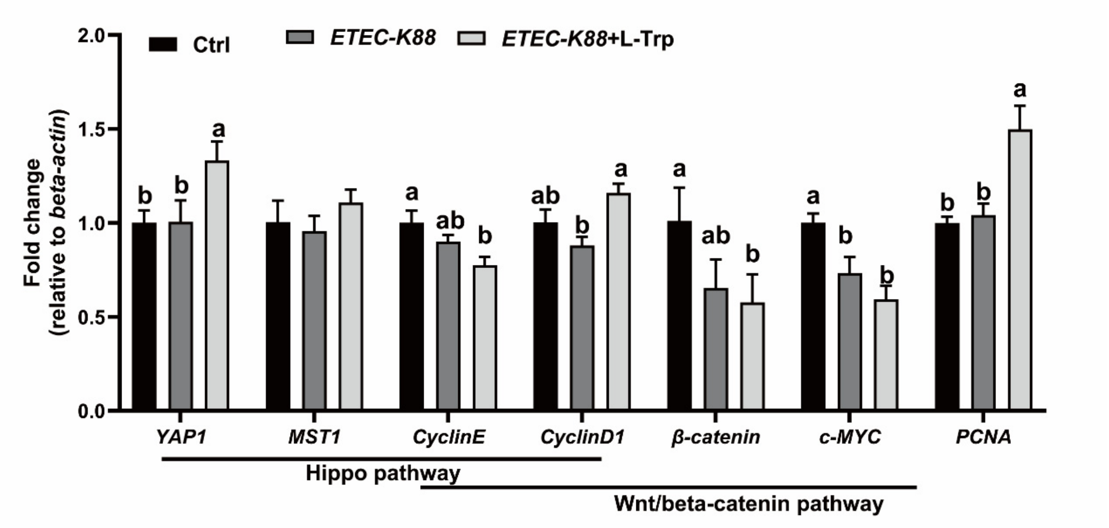

Figure S1. Effect of L-Trp on proliferation-related genes in ETEC-K88-infected IPEC-J2 cells

The expressions of IPEC-J2 with ETEC-K88 infection on the Hippo and Wnt/β-catenin signaling pathways. Data are presented as mean ± SEM. Different lowercase letters (a, b) above the bars indicate significant differences between groups ($P < 0.05$), while bars with the same letter (e.g., ab) are not significantly different.

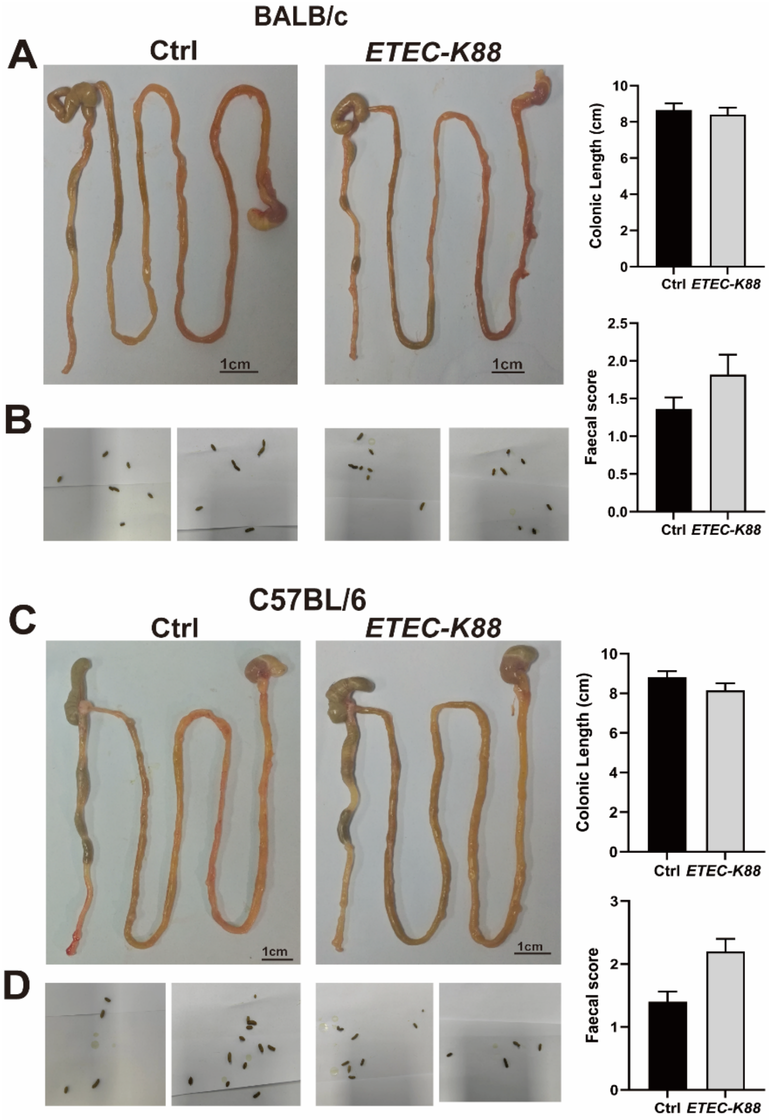

Figure S2. Effects of ETEC-K88 infection on intestinal morphology and fecal traits in different mouse strains.

(A, C) Gross morphological appearance of the isolated gastrointestinal tracts from BALB/c and C57BL/6 mice in the control (Ctrl) and ETEC-K88-infected groups, respectively. Eppendorf tubes are provided on the left as size references. **(B, D)** Representative fecal pellets from the corresponding groups. Mice in the control group exhibited normal granular fecal pellets, whereas mice in the ETEC-K88-infected groups displayed intestinal hyperemia, swelling of the intestinal wall, and reduced colon length.
